# Characteristics of Postoperative Patients with Breast Cancer Aged 65 Years and Older

**DOI:** 10.3390/curroncol30010052

**Published:** 2023-01-04

**Authors:** Yoshiteru Akezaki, Eiji Nakata, Masato Kikuuchi, Ritsuko Tominaga, Hideaki Kurokawa, Masaki Okamoto, Toshifumi Ozaki, Kenjiro Aogi, Shozo Ohsumi, Shinsuke Sugihara

**Affiliations:** 1 Division of Physical Therapy, Kochi Professional University of Rehabilitation, Kochi 781-1102, Japan; 2 Department of Orthopaedic Surgery, Okayama University Hospital, Okayama 700-8558, Japan; 3 Department of Rehabilitation Medicine, National Hospital Organization Shikoku Cancer Center, Ehime 791-0280, Japan; 4 Department of Breast Oncology, National Hospital Organization Shikoku Cancer Center, Matsuyama 791-0280, Japan

**Keywords:** breast cancer, elderly, comorbidity, upper-limb function, rehabilitation

## Abstract

Objective: This study aimed to compare postoperative patients with breast cancer aged ≥65 years with those aged <65 years and clarify the characteristics of postoperative patients with breast cancer aged ≥65. Methods: In total, 376 patients in whom we were able to evaluate survey items one month after surgery were included in the study. Comorbidity, including diabetes mellitus and hypertension, shoulder range of motion (ROM), upper-limb function, and psychological problems, was evaluated. Results: Hypertension and diabetes mellitus were significantly higher in patients aged ≥65 years (the elderly group) than in those aged <65 years (the non-elderly group) (*p* < 0.05). Preoperative shoulder flexion ROM was significantly restricted in the elderly group compared with the non-elderly group (*p* < 0.05). Preoperative shoulder abduction ROM was significantly restricted in the elderly group compared with the non-elderly group (*p* < 0.05). At one month after surgery, upper-limb function was more impaired in the non-elderly group than in the elderly group (*p* < 0.05). In both groups, both ROM and upper-limb function were significantly impaired one month after surgery compared with before surgery (*p* < 0.05). Conclusions: Postoperative patients with breast cancer aged ≥65 years should be careful about risk management and intervention during rehabilitation. Preoperative evaluation of shoulder ROM should be performed because patients aged ≥65 years have limited ROM before surgery.

## 1. Introduction

Breast cancer is the most common cancer in the world and among women [1]. Age is associated with cancer risk [2]. Breast cancer is more common in patients over the age of 65 years [3]. By 2030, approximately 70% of all cancers will be diagnosed in adults aged 65 years and older [2]. Early detection and improved treatment have increased the survival rate of affected women [4]. Therefore, the number of patients after cancer surgery and patients undergoing cancer treatment will increase.

Surgery plays a central role in treating early-stage breast cancer. Functional limitation of the upper body is one of the most common complications after breast cancer surgery. Breast cancer surgery has been reported to result in restricted shoulder range of motion (ROM), decreased upper-extremity strength, disabilities, such as pain, pectoralis stiffness, lymphedema, and axillary web syndrome, and impaired shoulder function [5,6,7,8,9]. Patients with breast cancer also develop psychiatric disorders, such as depression, anxiety, and decreased quality of life [10,11]. Preoperative and postoperative rehabilitation were performed in patients with breast cancer, and effects of improving shoulder ROM, upper-limb muscle strength, and Quality of life were reported [12,13].

Elderly people have comorbidities and functional decline [14]. A sizeable proportion of patients older than 70 years with operable breast cancer die of non-cancer-related causes [15]. Rehabilitation for elderly patients with breast cancer should be performed with an understanding of their characteristics, including not only physical function but also comorbidities. There are few studies, however, that have compared comorbidities, upper-extremity function, and psychological disorders between the elderly and non-elderly.

Therefore, this study aimed to compare postoperative patients with breast cancer aged ≥65 years and those aged <65 years and clarify the characteristics of postoperative patients with breast cancer aged ≥65 years.

## 2. Patients and Methods

The subjects in this study were 447 consecutive postoperative patients with breast cancer with axillary lymph node dissection at our hospital between November 2013 and March 2018. Among the 447 patients, 376 who were evaluable in the survey items one month after surgery were included in the study and the remaining, in whom it was difficult to evaluate survey items one month after surgery, were excluded. All subjects were female. In this study, patients were classified into patients aged ≥65 years (elderly group) and those aged <65 years (non-elderly group). Body mass index (BMI), neoadjuvant chemotherapy (yes, no), postoperative chemotherapy (yes, no), postoperative hormonal therapy (yes, no), postoperative radiotherapy (yes, no), comorbidity, shoulder ROM, upper-limb function, and psychological problems were evaluated.

### 2.1. Rehabilitation Program

Preoperative rehabilitation was performed during hospitalization and we provided guidance on upper-limb exercises. Postoperative rehabilitation was started on the first and second days after the operation, and exercise was performed distal to the elbow joint. Shoulder ROM exercise was performed depending on the pain claimed by patients within 90° of shoulder joint flexion and abduction on the third day after the operation to the day of drain withdrawal. After drain removal, upper-limb ROM exercise was performed without restriction of shoulder ROM. After discharge, the patients were instructed to perform upper-limb exercise therapy.

### 2.2. Comorbidity

The presence of diabetes mellitus, hypertension, and cardiac morbidity was investigated. Patients had received a physician-confirmed evaluation of diabetes mellitus, hypertension, and cardiac morbidity before surgery. Cardiac morbidity included angina pectoris, congestive heart failure, or valvular heart disease.

### 2.3. Psychological Problems

The presence of psychological disorders was evaluated using the Distress and Impact Thermometer (DIT). DIT is a two-item self-reported questionnaire for assessing patients’ psychological distress on an 11-point Likert-type scale, ranging from 0 (not at all) to 10 (extremely) [16]. The standard cut-off scores for screening for adjustment disorders, depression, and major depression with suicidal ideation are a “distress” score of 4 or above and an “impact” score of 3 or above, a “distress” score of 5 or above and an “impact” score of 4 or above, and a “distress” score of 5 or above and an “impact” score of 5 or above, respectively. In this study, patients were classified into the following two groups using DIT: the psychological problem group with adjustment disorders, depression, or major depression and suicidal ideation in the psychological problem group and the non-psychological problem group.

The measurements were performed before surgery and one month after surgery.

### 2.4. Shoulder ROM Test

The shoulder ROM test of active flexion and abduction was performed by an occupational therapist and physical therapist using a goniometer.

The evaluation of shoulder ROM was performed before surgery and one month after surgery.

### 2.5. Upper-Limb Function

Upper-limb function was evaluated using the Disability of Arm, Shoulder, and Hand (DASH). DASH is a self-administered questionnaire that evaluates symptoms and physical function of the upper limb [17].

The DASH was assessed before surgery and one month after surgery.

### 2.6. Statistical Analysis

Comparisons between before and one month after surgery items and improvement rates were performed using the chi-square and Mann–Whitney U tests.

A comparison of the survey items before surgery and one month after surgery was performed using the Wilcoxon signed-rank and McNemar tests.

Data analysis was performed using SPSS software version 22.0 (IBM, Tokyo, Japan). The results were considered significant when the probability of error was less than 5% (*p* < 0.05).

## 3. Results

### 3.1. Comorbidity

Table 1 shows the differences in BMI, treatment, and comorbidity between the elderly and non-elderly groups. The elderly group received neoadjuvant and postoperative chemotherapy significantly less frequently than the non-elderly group (*p* < 0.05). The overall proportion of patients with hypertension was 9.3% (35/376), 16.9% (14/83) in the elderly group and 7.1% (21/293) in the non-elderly group (*p* < 0.05).0 Hypertension and diabetes mellitus were significantly higher in the elderly group than in the non-elderly group (*p* < 0.05). The overall proportion of patients with diabetes mellitus was 4.8% (18/376), 10.8% (9/83) in the elderly group and 3.1% (9/293) in the non-elderly group (*p* < 0.05).

### 3.2. Psychological Problems

There are no significant differences in psychological problems between the elderly group and younger patients, preoperatively (Table 2).

No significant change was observed before surgery and one month after surgery in both the elderly group and the non-elderly group (Table 2).

### 3.3. ROM and DASH

Table 2 shows the differences in ROM test and DASH between the elderly and non-elderly groups.

Preoperative shoulder flexion ROM was significantly restricted in the elderly group (average 159 degrees) compared with the non-elderly group (average 163.7 degrees) (*p* < 0.05). Preoperative shoulder abduction ROM was significantly restricted in the elderly group (average 160.3 degrees) compared with the non-elderly group (average 164.9 degrees) (*p* < 0.05). At one month after surgery, DASH was more impaired in the non-elderly (average 9.0 scores) group than in the elderly group (average 8.5 scores) (*p* < 0.05).

In both groups, both ROM and DASH were significantly impaired one month after surgery compared with before surgery (*p* < 0.05).

The difference in shoulder flexion between before surgery and one month after surgery was −19.3 ± 17.4° in the elderly group and −22.7 ± 19.1° in the non-elderly group. Shoulder abduction was −25.4 ± 26.7° in the elderly group and −29.9 ± 26.5° in the non-elderly group. DASH score was 9.2 ± 15.2 in the elderly group and 12.7 ± 15.7 in the non-elderly group. The DASH value was significantly higher in the non-elderly group than in the elderly group, and the upper-limb function was significantly worse in the non-elderly group (*p* < 0.05).

## 4. Discussion

In this study, the differences in comorbidity, ROM, and upper-limb function between the elderly and the non-elderly groups were examined. The study results showed that the elderly group had significantly more patients with diabetes mellitus and hypertension than the non-elderly group. Preoperative shoulder ROM was significantly restricted in the elderly group compared with the non-elderly group. At one month after surgery, upper-limb function was significantly impaired in the non-elderly group compared with the elderly group.

Previous studies reported that 16–20% of breast cancer subjects are diagnosed with type 2 diabetes mellitus [18,19]. Patients with diabetes may present at the time of cancer treatment with already impaired physical function levels associated with their diabetes mellitus [20,21]. Breast cancer patients with diabetes mellitus are at an increased risk of developing chemotherapy-induced neuropathy [22,23] and chemotherapy-related toxicity [18]. In this study, the proportion of patients with diabetes was significantly higher among patients with breast cancer aged ≥65 years than among those aged <65 years. Combining aerobic exercise with resistance exercise is effective for patients with diabetes [24,25]. Therefore, patients with breast cancer with diabetes mellitus need appropriate intervention in addition to upper-limb ROM exercise after surgery. Patients with type 2 diabetes mellitus have shown an association between the degree of hyperglycemia and increased risk of myocardial infarction [26,27,28] and stroke [29]. This suggests that even a modest reduction in glycemia has the potential to prevent death from complications related to diabetes mellitus [30,31]. Cancer patients with known diabetes mellitus will require close monitoring of blood glucose concentrations with the appropriate intervention depending on their baseline diabetic management.

Nechuta et al. [32] revealed that the main comorbidity in patients with breast cancer was hypertension (22.4%). Kozłowska et al. showed that 74% of patients with breast cancer aged ≥65 years had hypertension [33]. In this study, the proportion of patients with hypertension was 4.8%, lower than in previous studies, but there were significantly more patients with hypertension aged ≥65 years with breast cancer. Hypertension is an important risk factor in stroke development [34,35]. Patients with hypertension require medical management, including medication. Additionally, in providing rehabilitation, exercise therapy should be performed while managing risk by measuring blood pressure. Our study found that elderly patients with breast cancer are more likely to have diabetes and hypertension than non-elderly patients; thus, special attention needs to be paid to those aspects during rehabilitation interventions.

Box et al. [36,37] and Gutman et al. [38] demonstrated good shoulder motion outcomes from 3 to 24 months after surgery. Older patients (65–80 years old) have a limited shoulder ROM compared with young patients (20–35 years old) [39]. In this study, both the elderly and non-elderly groups showed a significant decrease one month after surgery compared with before surgery. Furthermore, no significant difference in the improvement rate was observed between the two groups. Shoulder ROM showed no difference in improvement with age until one month after surgery. However, preoperative ROM showed significantly lower results in the elderly group than in the non-elderly group. Decreases in ROM across age are affected by changes in the musculoskeletal system, such as reduced elasticity of the ligaments, reductions in cartilage resilience, and decreased muscle strength [40,41]. The decreased ROM in the shoulder in the elderly before surgery is associated with musculoskeletal changes across age. Exercise therapy is performed to improve upper-limb function due to postoperative ROM limitation of the shoulder joint and its effect has been reported [42]. It was also found that shoulder ROM is restricted for patients, which suggests the need for shoulder ROM evaluation for elderly patients with breast cancer before surgery.

Previous studies reported that 55.4–62% of women had some level of upper-limb dysfunction [43,44]. There are few studies, however, that have compared upper-extremity function between the elderly and non-elderly. In this study, DASH did not show a significant difference between the two groups before surgery, but a significantly higher degree of disability was observed one month after surgery in the elderly group than in the non-elderly group. Additionally, the improvement rate showed that the non-elderly group had a significantly higher degree of disability than the elderly group. Compared with the elderly group, the non-elderly group is more likely to use the upper limbs, and postoperatively, ROM restrictions often cause disorders in the movement of the upper limbs. The number of patients with upper-limb dysfunction is higher in the non-elderly group than in the elderly group, so guidance for ADL using the upper limbs is required.

Several studies demonstrated that elderly patients are less likely to receive chemotherapy [45,46,47]. In this study, we also showed that significantly fewer patients in the elderly group received chemotherapy than those in the non-elderly group. Patients aged ≥65 years have fewer opportunities to visit the hospital regularly after discharge, and healthcare workers may have difficulty in verifying patient status. Therefore, healthcare workers must make efforts to regularly evaluate the condition of the elderly, even after they are discharged from the hospital.

## 5. Study Limitations

This study had some limitations. First, this study was a single-center study and multicenter research was not conducted. Particularly, comorbidities may differ between hospitals and regions. Second, physical functions are only ROM and upper-limb function, and muscle strength, balance, endurance, and so on were not evaluated. Thus, further research is needed to examine these factors.

## 6. Conclusions

Rehabilitation of postoperative patients with breast cancer aged ≥65 years requires careful management of risk, not only for upper-extremity function but also for comorbidities. Further, preoperative evaluation of shoulder ROM should be performed because patients aged ≥65 years have limited ROM before surgery.

## Figures and Tables

**Table 1:** Comparison of BMI, treatment, and comorbidity between the elderly and non-elderly groups.

Variable	Elderly Group (*n* = 83)	Non-Elderly Group (*n* = 293)	*p*-Value
BMI (kg/m^2^) ^a^	23.6 ± 3.6	23.2 ± 4.2	0.101
Neoadjuvant chemotherapy ^b^			0.003
Yes	11	87	
No	72	206	
Postoperative chemotherapy ^b^			*p* < 0.0001
Yes	26	166	
No	57	127	
Postoperative hormonal therapy ^b^			1.000
Yes	32	114	
No	51	179	
Postoperative radiotherapy ^b^			0.547
Yes	32	114	
No	51	179	
Diabetes mellitus ^b^			0.007
Yes	9	9	
No	74	284	
Hypertension ^b^			0.017
Yes	14	21	
No	69	272	
Cardiac morbidity ^b^			0.741
Yes	2	11	
No	81	282	

^a^ Mean ± standard deviation; ^b^ number. BMI, body mass index.

**Table 2:** Comparison of ROM-T, DASH, and DIT between the elderly and non-elderly groups.

Variable	Elderly Group (*n* = 83)			Non-Elderly Group (*n* = 293)				
	Before	1 Month	*p*-Value ^c^	Before	1 Month	*p*-Value ^d^	*p*-Value ^e^	*p*-Value ^f^
Shoulder flexion ROM-T (degrees) ^a^	159.0 ± 15.9	140.0 ± 17.8	*p* < 0.0001	163.7 ± 13.4	140.9 ± 20.4	*p* < 0.0001	0.021	0.395
Shoulder abduction ROM-T (degrees) ^a^	160.3 ± 17.1	134.9 ± 26.2	*p* < 0.0001	164.9 ± 14.6	135.0 ± 26.9	*p* < 0.0001	0.024	0.986
DASH (scores) ^a^	8.5 ± 10.1	17.7 ± 15.1	*p* < 0.0001	9.0 ± 13.4	21.7 ± 14.1	*p* < 0.0001	0.274	0.003
DIT ^b^								
PPG	34	32	0.864	135	121	0.185	0.454	0.705
NPPG	49	51		158	172			

^a^ Mean ± standard deviation, ^b^ number, ^c^ before surgery vs. 1 month after surgery in the elderly group, ^d^ before surgery vs. 1 month after surgery in the non-elderly group, ^e^ before surgery in the elderly group vs. before surgery in the non-elderly group, and ^f^ 1 month after surgery in the elderly group vs. 1 month after surgery in the non-elderly group; ROM-T, range of motion test; DASH, Disabilities of the Arm, Shoulder, and Hand; DIT, Distress and Impact Thermometer; PPG, psychological problem group; NPPG, non-psychological problem group.

## Data Availability

The data presented in this study are available on request from the corresponding author. The data are not publicly available due to participants’ personal information.
